# Coelenterazine sulfotransferase from *Renilla muelleri*

**DOI:** 10.1371/journal.pone.0276315

**Published:** 2022-10-17

**Authors:** George Tzertzinis, Brenda Baker, Jack Benner, Elizabeth Brown, Ivan R. Corrêa, Laurence Ettwiller, Colleen McClung, Ira Schildkraut

**Affiliations:** New England Biolabs, Ipswich, Massachusetts, United States of America; Anhui Polytechnic University, CHINA

## Abstract

The luciferin sulfokinase (coelenterazine sulfotransferase) of *Renilla* was previously reported to activate the storage form, luciferyl sulfate (coelenterazine sulfate) to luciferin (coelenterazine), the substrate for the luciferase bioluminescence reaction. The gene coding for the coelenterazine sulfotransferase has not been identified. Here we used a combined proteomic/transcriptomic approach to identify and clone the sulfotransferase cDNA. Multiple isoforms of coelenterazine sulfotransferase were identified from the anthozoan *Renilla muelleri* by intersecting its transcriptome with the *LC-MS/MS* derived peptide sequences of coelenterazine sulfotransferase purified from *Renilla*. Two of the isoforms were expressed in *E*. *coli*, purified, and partially characterized. The encoded enzymes display sulfotransferase activity that is comparable to that of the native sulfotransferase isolated from *Renilla reniformis* that was reported in 1970. The bioluminescent assay for sensitive detection of 3’-phosphoadenosine 5’-phosphate (PAP) using the recombinant sulfotransferase is demonstrated.

## Introduction

**Fig 1:**
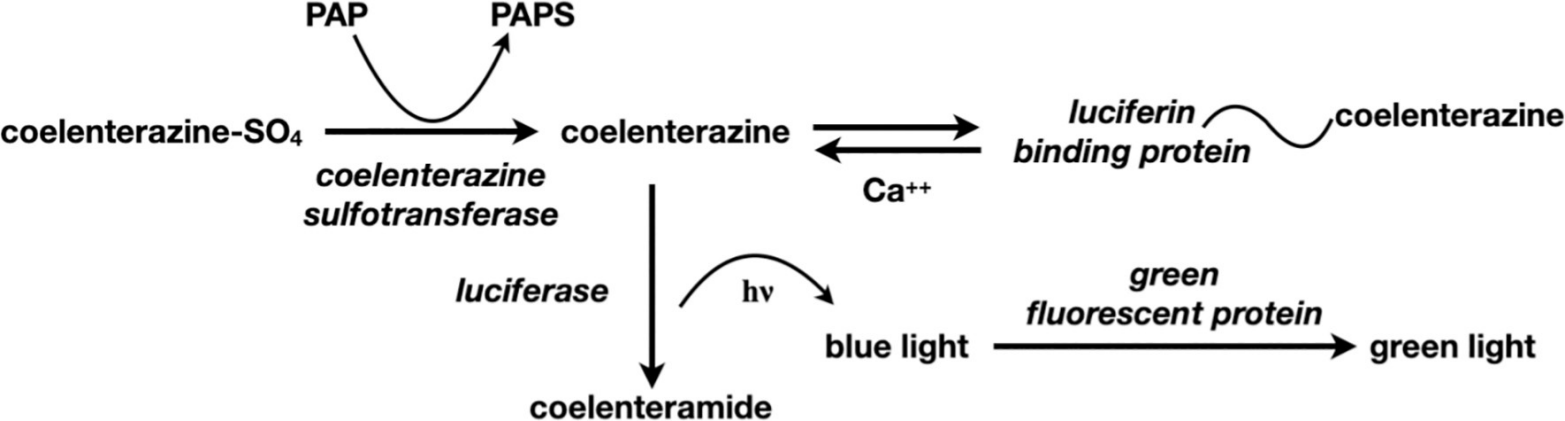
*Renilla* bioluminescence pathway.

Several genes involved in bioluminescence have been cloned from the marine anthozoan, *Renilla* (Fig 1). These genes encode the green fluorescent protein[1] luciferin binding protein [2], and luciferase (RLuc)[3].

**Fig 2:**
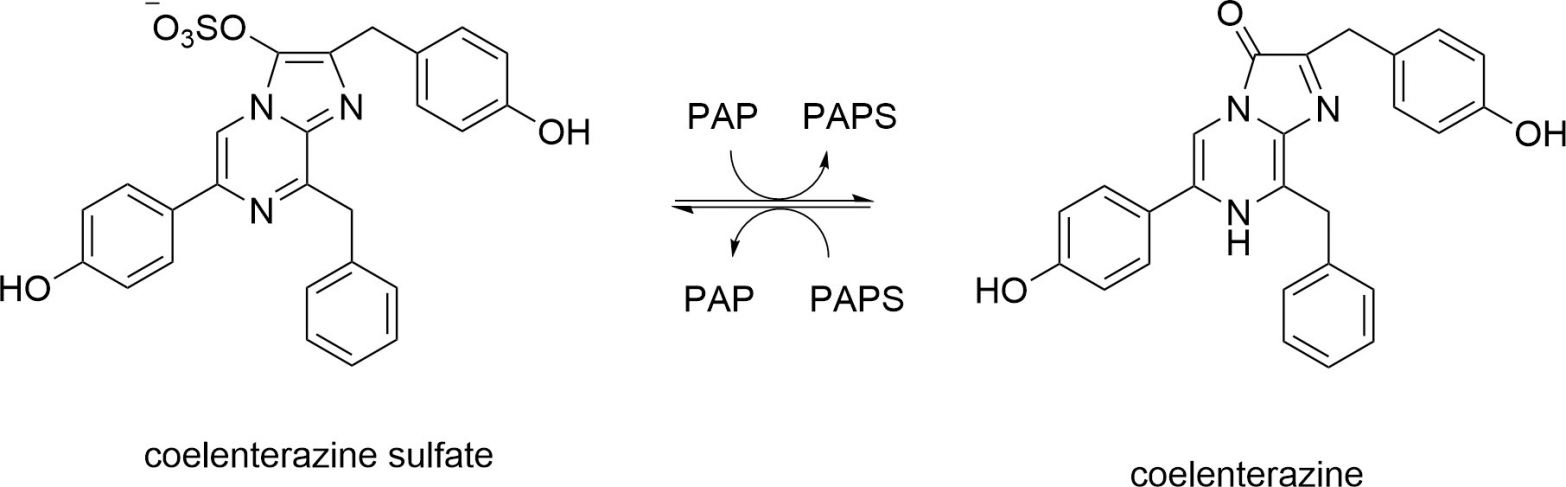
Structures of coelenterazine and coelenterazine sulfate and their interconversion.

The availability of these proteins has been facilitated by their expression from recombinant sources. An additional enzyme involved in the bioluminescence pathway is the luciferin sulfokinase (coelenterazine sulfotransferase) which activates the storage form of the marine luciferin, coelenterazine sulfate. In 1970 Cormier *et al*. [4] reported the isolation of the sulfokinase from *R*. *reniformis* and demonstrated that the enzyme preparation could convert luciferyl sulfate into luciferin and also catalyze the reverse reaction, luciferin to luciferyl sulfate. The chemical structure of *Renilla* luciferin was determined by Hori *et al*. 1977 [5] and Shimomura and Johnson 1975 [6] and the substrate was termed coelenterazine Fig 2.

The luciferin sulfokinase was shown to transfer the sulfuryl group from the storage form, luciferyl sulfate (coelenterazine sulfate), to 3’-phosphoadenosine 5’-phosphate (PAP) to form 3’-phosphoadenosine 5’-phosphosulfate (PAPS) and luciferin (coelenterazine) (Fig 2). In this study, we refer to luciferin sulfokinase as coelenterazine sulfotransferase (Coel-ST).

**Fig 3:**
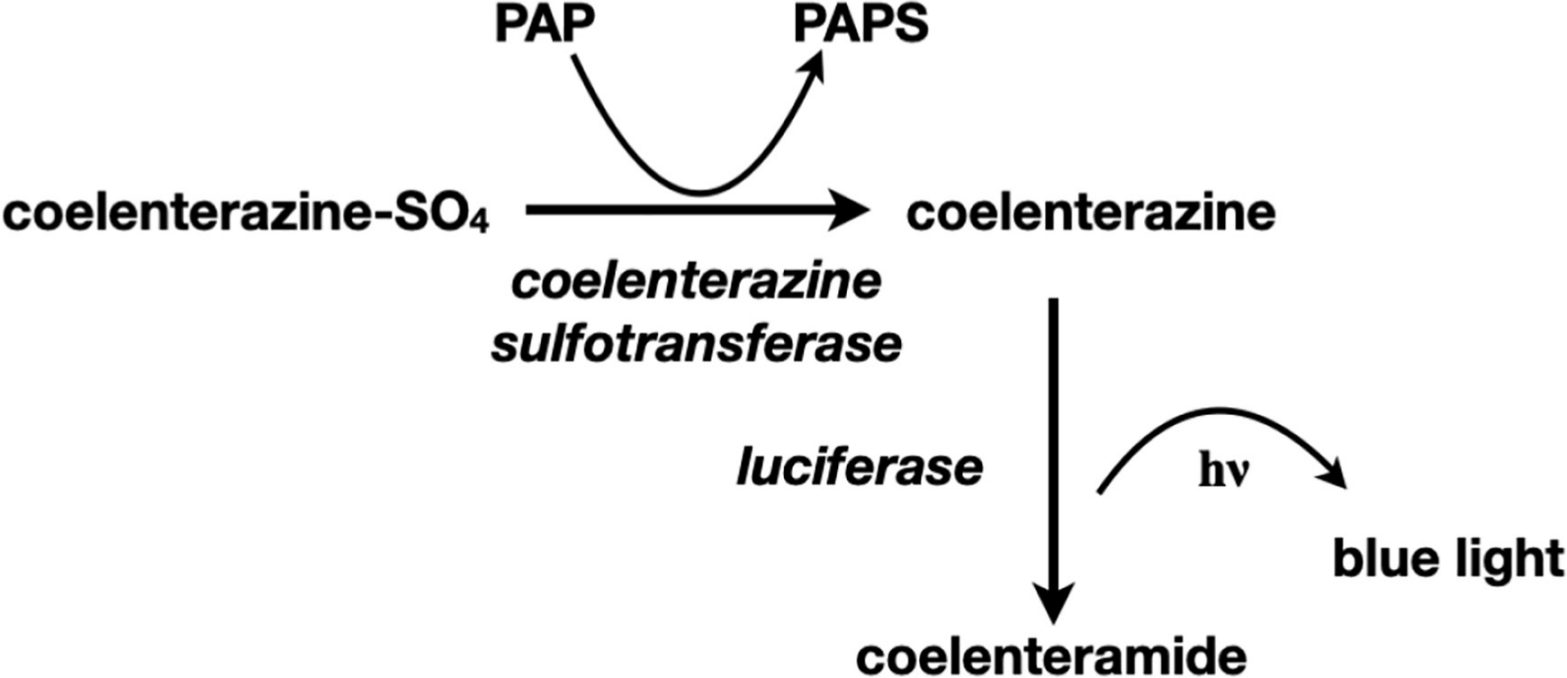
Cascade for conversion of coelenterazine sulfate to coelenteramide with concomitant emission of light utilizing PAP as the sulfate acceptor.

The native Coel-ST enzyme has been used to detect PAP in an assay described by Stanley in 1975 [7] and refined further by Anderson in 1978 [8], and remains a useful highly sensitive assay for PAP (Fig 3). However, the difficulty of procuring this sulfotransferase from the native source has prevented the use of this assay to measure low levels of PAP in different disease states and tissues. There exists today a need for using the very sensitive PAP assay of Stanley/Anderson, especially as PAP/PAPS is a substrate for many metabolic reactions. For example, measuring PAP concentration in tissues could be informative for patients who undergo lithium treatment, because PARP-1 activity in cells is strongly inhibited by higher PAP levels, which reduces the cells ability to repair DNA damage [9]. The absence of the recombinant form of this protein has been a barrier to its structural and biochemical characterization and its application in linked biochemical assays.

Here we report the cloning, expression, amino acid sequence, and purification of the recombinant form of the *Renilla muelleri* Coel-ST and an update to the synthesis of coelenterazine sulfate [10]. We show that the recombinant form of Coel-ST can be used in preference to the native enzyme making accessible the PAP assay described by Stanley [7].

## Materials and methods

### Purification and molecular weight determination of native *Renilla* coelenterazine sulfotransferase

The *R*. *muelleri* were purchased from Gulf Specimen Marine Laboratories, Panacea, FL. The initial steps in the purification followed the method of Anderson [8] through homogenization and filtering. The coelenterazine sulfotransferase was purified from 800 grams tissue, corresponding to 100 live animals. The enzyme was purified, guided by measuring sulfotransferase activity, through the following series of chromatographic steps, Q Sepharose, S200 gel sieving, Source Q, DEAE Hyper D, and TSK Heparin. The most active fraction eluting from the Heparin column (2 ml volume) was concentrated to 50 μl by ultrafiltration spin column and corresponds to the final enzyme preparation (S1 Fig) used for peptide sequencing and characterization.

For molecular weight determination, a sample of the pool of active fractions obtained from the Source Q chromatographic step was applied to a Superdex 75 column calibrated with molecular weight standards. The fractions containing the sulfotransferase activity were coincident with the *E*. *coli* maltose binding protein with a molecular weight of 42 kD.

### Peptide sequencing by 2D LC-MS/MS

The purified Coel-ST sample was subjected to proteolytic digestion using a Filter-Aided Sample Preparation Kit (Abcam, Cambridge, UK) [11]. The sample was reduced, alkylated with iodoacetamide, and digested on-filter using 5 μg of Trypsin-ultra™M Mass Spectrometry Grade (New England Biolabs, Ipswich, MA) overnight at 37°C. The resultant peptide mixture was acidified with formic acid to 0.1% for mass spectrometric analysis. The mixture was analyzed using a two-dimensional LC MS/MS system consisting of a Dionex UltiMate 3000 UHPLC and a Q Exactive mass spectrometer (Thermo Fisher Scientific, Waltham, MA). The peptides were loaded onto a self-packed SCX precolumn (5 cm x 5 μ Luna SCX, Phenomenex, Torrance, CA), pushed with two salt pulses onto a self-packed RP analytical column (15 cm x 5 μ Luna C18, Phenomenex), and eluted over two-hour reverse phase separation gradients. Peptides were introduced to the mass spec by nano electrospray, and precursor peptides between 400–1600 m/z (FT resolution 70,000) were selected for fragmentation. MS/MS fragmentation of the Top 10 precursor ions was conducted with data-dependent settings. Dynamic exclusion was set at 15 seconds and no charge states were rejected.

The MS/MS spectra were analyzed using PEAKS with semi specific tryptic cleavage, a precursor mass tolerance of 10 ppm, fragment ion tolerance of 0.02 Da, and up to two missed cleavages. The spectra were searched against a custom fasta database consisting of proteins translated from the *R*. *muelleri* transcriptome.

### *Renilla* RNA transcriptome

*Renilla* total RNA was prepared from 2 live animals by freezing in liquid nitrogen, homogenization by mortar and pestle, and Trizol extraction. Poly-A^+^ RNA was isolated from 600 μg total RNA using the poly-A Spin mRNA isolation kit from New England Biolabs (NEB) using two consecutive oligo-dT selections following the kit protocols. 50 ng of poly-A^+^ RNA was used to construct a directional cDNA library using the NEBNext Ultra RNA library kit for Illumina following the kit instructions. The fragmentation step was performed for 13 min at 94°C. Final amplification was performed for 15 cycles and the library was purified from primers and adaptors using two consecutive Ampure bead steps before quality assessment in an Agilent Bioanalyzer which indicated a library with a peak corresponding to 262 bp.

Sequencing of the library was performed in the Illumina MiSeq. 12 million reads were obtained.

The reads were used as input for the Trinity assembly software [12] for *de novo* transcript assembly, resulting in 48,880 assembled RNA transcripts. The corresponding DNA sequences were generated. The entire assembled cDNA library generated from Trinity was translated in all 3 frames.

### Cloning of the Coel-ST Cdna

Poly-A^+^ RNA was used to generate cDNA with the Protoscript II First strand cDNA synthesis kit (NEB) and random primers. The cDNA was used as a template for PCR amplification with forward (F) and reverse (R) primers designed on the two open reading frames (ORF) 12701 and ORF 16712 respectively, with Q5 High-Fidelity 2X Master Mix (NEB), in order to generate a “full length” cDNA corresponding to the ORF 12.16. The primers included restriction sites to facilitate cloning into the expression vector pET28a.

Primers:

12701-F: CCA ATT CGC ATA TGT CTG CGA CTA AAG AAA CAC AAT CTT C

16712-R: ATA GGA TCC TTA ATA TTT CAA AGG ACA TTC TTC TCG TAT AGC ATA C

### Purification of recombinant coelenterazine sulfotransferases

The Coel-ST cDNAs from isoforms 3A and 4A were cloned between the NdeI and BamHI sites of kanamycin resistant (kanR) pET28a, which resulted in amino terminal his tagged proteins. The resulting kanR plasmids were transformed into T7 Express *E*. *coli* (NEB c2566 competent cells). The clones were grown in one liter of LB media at 37°C until ~0.5 OD600 units, IPTG was added to a final concentration of 0.3 mM and the cultures were incubated at 16°C overnight. The cultures were harvested by centrifugation and the cell pellets were suspended in 40 mM Tris-HCl pH 7.5, 1 M NaCl, 40 mM imidazole, 2 mM DTT and 10% glycerol. The cells were lysed at 4°C by sonication and clarified by centrifugation. The supernatants were applied to an IMAC column, washed and eluted with an imidazole gradient from 40 mM to 500 mM in the above buffer. The peak of activity eluted mid-gradient and was pooled and dialyzed against 20 mM Tris-HCl pH 8.0, 200 mM NaCl, 1 mM DTT, 0.1 mM EDTA and 50% glycerol.

### Preparation of coelenterazine sulfate

**Fig 4:**
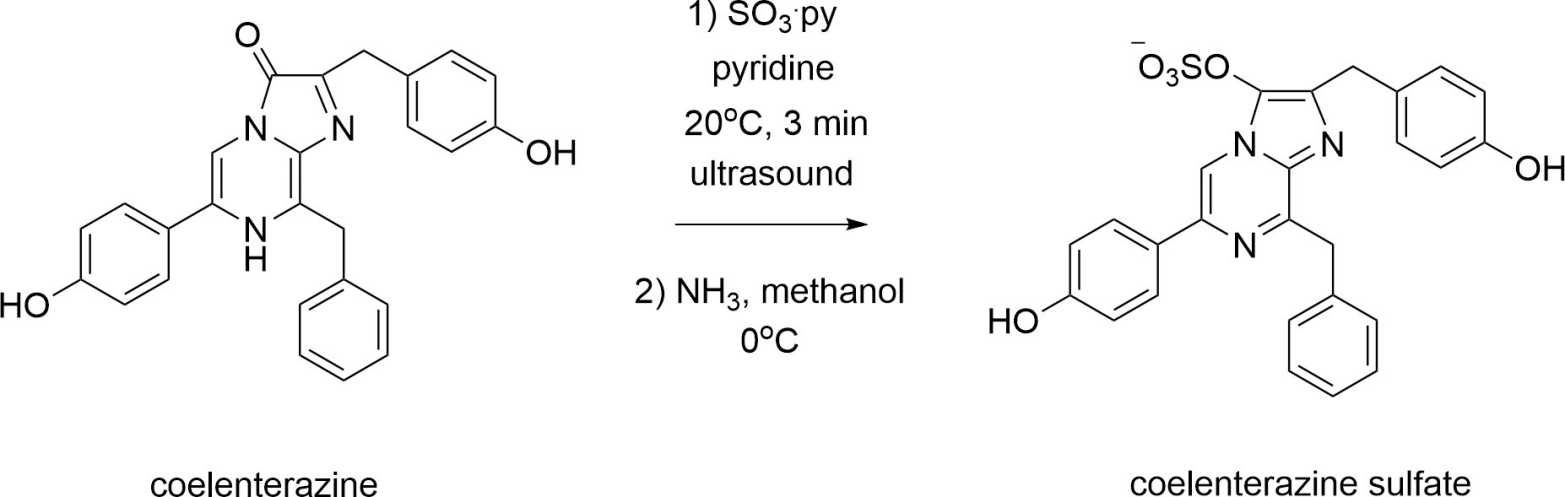
Scheme for coelenterazine sulfate chemical synthesis.

Coelenterazine sulfate was prepared by reacting coelenterazine (Gold Biotechnology, #CZ10) with sulfur trioxide according to protocol adapted from Teranishi, K. 2002 [10]. Commercially available compounds were obtained from Sigma-Aldrich (unless specified otherwise) and used without further purification. Briefly, coelenterazine (2.0 mg, 4.7 μmol) was dissolved in anhydrous pyridine (0.5 mL) and sulfur trioxide pyridine complex (4.0 mg, 25.1 μmol) was added. The reaction mixture sonicated in an ultrasonic bath at 20°C for 3 min (protected from light). The reaction completion was monitored by LC/MS on Agilent 6120 Single Quad System 1200 Series equipped with a Waters Atlantis T3 C18 column (2.1 x 150 mm, 5 μm particle size). After completion, the reaction mixture was poured into a 2 M ammonia solution in methanol (0.5 mL) at 0°C. The solvent was removed under vacuum at 20°C. The crude product was redissolved in 1:9 acetonitrile/water (2 mL) and purified by reversed-phase HPLC on an Agilent 1100 Preparative-scale Purification System equipped with a VYDAC 218TP series C18 polymeric reversed-phase column (22 x 250 mm, 10 μm particle size) at a flow rate of 20 mL/min and using an acetonitrile/water gradient of 5–95% over 40 min (absorbance was monitored at 263 and 433 nm). ESI-MS [M]^−^*m/z* 502.1 (calc. for C_26_H_20_N_3_O_6_S^–^ *m/z* 502.1078). The yield after HPLC purification was 55% (Fig 4).

### RLuc preparation

*Renilla* luciferase (RLuc) was prepared as described in Tzertzinis [13] with an additional Source Q chromatography step to remove contaminating phosphatase activity.

### Coelenterazine sulfotransferase assay

The assay used for coelenterazine sulfotransferase activity is based on the assay of Cormier [14]. The assay is linked to the bioluminescent *Renilla* luciferase reaction. It requires conversion by the sulfotransferase of coelenterazine sulfate and PAP to coelenterazine and PAPS respectively and subsequent conversion of coelenterazine to coelenteramide by the *Renilla* luciferase (Fig 3).

Typically, a reaction mixture containing 100 mM Bis-tris propane pH 7.0, 1 mM DTT, 1 μM coelenterazine sulfate, 20 μM PAP, 10 nM RLuc was first incubated at room temperature for 5 minutes to reduce the background luminescent signal which emanated from trace coelenterazine present in the coelenterazine sulfate. This reaction mixture was then aliquoted at 100 μl per well into 96 well plates. The plates were placed into a Centro LB 960 luminometer (Berthold) plate reader, set at 25o C, and each well was first read for 0.5 seconds at 5 second intervals for one minute to establish that the background signal was stable. The sulfotransferase reaction was initiated by the addition of Coel-ST and activity monitored by reading each well for 0.5 seconds every 5 seconds for up to 30 minutes. The luminescence generated by the reaction overtime is constant and is proportional to the amount of sulfotransferase. The activity is reported as relative light units per 1 second (RLU/s).

### PAP assay

T4 polynucleotide kinase (3’-phosphatase minus, M0236) was obtained from New England Biolabs (NEB). 3’-phosphoadenosine 5’-phosphate and adenosine 3’-monophosphate were from Sigma-Aldrich. 100 μl reactions containing 100 mM Bis-tris propane pH 7.0, 1 mM DTT, 1mM EDTA, 200 nM coelenterazine sulfate, 10 nM of RLuc, and 1–20 nmoles of Coel-ST were performed at 25°C. Each reaction was initiated with the addition of the PAP sample and was followed by light emission. Relative light units per second (RLU/s) were measured in a Centro LB 960 luminometer (Berthold) plate reader.

## Results

**Fig 5:**
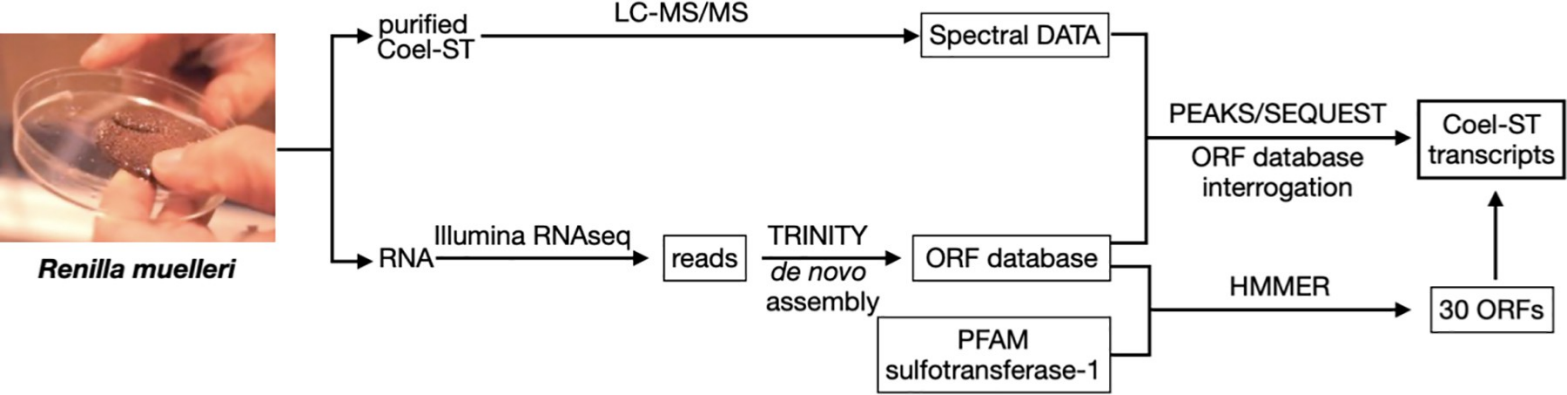
Outline of the hybrid approach followed for identification of the Coel-ST transcripts.

The strategy to obtain the recombinant form of *Renilla* Coel-ST, a combination of a proteomic and a transcriptomic approach, is outlined in Fig 5. We first isolated the native protein from *Renilla* and determined the sequences of the peptides that comprise it by 2D LC-MS/MS [15,16]. In parallel, we isolated *Renilla* RNA from which we derived and analyzed the *Renilla* transcriptome in the absence of any genomic sequence information. Using HMMER [17] we identified ORFs from the transcriptome which belong to PFAM-defined sulfotransferases (Family: *Sulfotransfer_1*). In addition, ORFs were interrogated with peptide sequences identified from 2D LC-MS/MS[18] (Fig 5). As described in detail below, an ORF was ultimately identified which matched the peptide sequences derived from the purified enzyme. cDNAs were expressed and the resulting recombinant enzyme converted coelenterazine sulfate to coelenterazine in a PAP dependent manner.

### Activity and size of the native coelenterazine sulfotransferase

The native sulfotransferase exhibited about 50-fold higher activity in the presence of added PAP than in its absence (S2 Fig), confirming the dependence on PAP and providing further evidence that the activity purified is a sulfotransferase and clearly not a sulfatase.

The molecular weight of the sulfotransferase was estimated at approximately 42 kD as determined by chromatographic gel sieving. This size is consistent with sulfotransferase-1 family members.

### Peptide sequencing by mass spectrometry

Using the purified protein, we proceeded to obtain peptide sequences by mass spectrometry. Ten microliters (20%) of the final sulfotransferase protein preparation were electrophoresed by SDS-PAGE. A gel slice corresponding to the region of 30 to 45 kD was excised from the gel, specifically excluding the 27 and 50 kD contaminant bands (S1 Fig) and subjected to proteolysis. The resulting peptides were subjected to analysis by 2D LC-MS/MS. The resulting spectral data were used as input with the program SEQUEST [19] to identify candidate ORFs corresponding to isolated Coel-ST as well as input for PEAKS v. 7.5 (Bioinformatics Solutions Inc., Waterloo, Ontario, Canada) to identify *de novo* peptide sequences. Approximately 101,000 spectra were obtained which represented 85,000 peptide sequences.

### cDNA library and transcriptome assembly

In order to obtain the Coel-ST coding sequence we generated a directional cDNA library from *R*. *muelleri* using Poly-A^+^ RNA as described in Materials and Methods. The sequences of the reads from this library were used for *de novo* assembly of the transcriptome in the absence of a reference genome using the Trinity software package [12]. The raw and processed sequencing data generated in this study have been submitted to the NCBI BioProject database (https://www.ncbi.nlm.nih.gov/bioproject/) under accession number PRJNA854012. The Trinity assembly yielded 48,880 putative transcript sequences. The entire collection of assembled cDNA sequences was translated in all 3 frames. The resulting protein sequences (ORFs) were used for identifying the Coel-ST sequence by intersecting two different approaches. First, they were used as input for the program HMMER [17] to identify sequence matches to a sulfotransferase profile-HMM, and second, as input for the program SEQUEST to screen the peptide sequences obtained by mass spectrometry analysis from the purified enzyme preparation. The two approaches are detailed below.

### HMMER analysis of the translated transcriptome

HMMER [17] was used to identify ORFs from the transcriptome which are associated with Family: *Sulfotransfer_1* [20]. This family includes a range of sulfotransferase proteins including flavonol 3-sulfotransferase, aryl sulfotransferase, alcohol sulfotransferase, estrogen sulfotransferase, and phenol-sulfating phenol sulfotransferase. We reasoned that the Coel-ST would belong to this family of STs. 31 ORFs were identified from the assembled *Renilla* transcripts which had E values ranging from 1.2e-37 to 0.34. Among the 31 ORFs was a small ORF of 37 amino acids, ORF 12701, and an ORF of 258 amino acids, ORF 16712. We noticed that the carboxy terminal 6 amino acids of ORF 12701 were identical to the 6 amino terminal amino acids of ORF 16712. The overlap of the 2 transcripts is 19 nucleotides. Trinity did not combine these two ORFs because the minimum overlap threshold for RNA assembly was set to the default value of 20 nucleotides. We manually assembled these 2 ORFs and named the result, ORF 12.16, reducing the total number of candidate ORFs to 30. This is a reasonable number, given that multiple sulfotransferases are responsible for catalyzing many different reactions in an organism [21]. In summary we identified 30 different putative sulfotransferases from the transcriptome and focused our attention to the ones most likely to use coelenterazine sulfate as a substrate.

### Identification of candidate ORFS for Coel-ST using MS-derived peptide sequences

**Fig 6:**
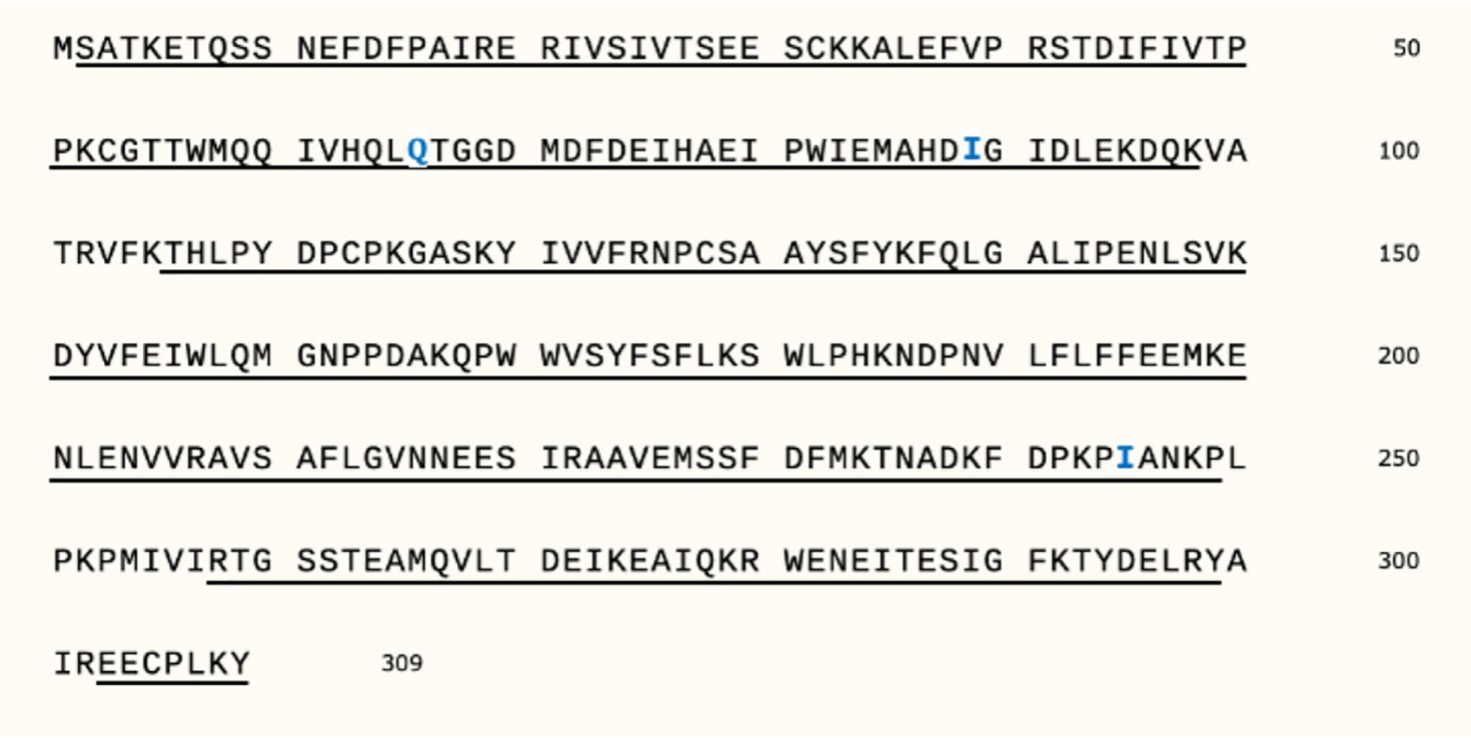
The *Renilla* coelenterazine sulfotransferase (Coel-ST) amino acid sequence from ORF 12.16 and LC-MS/MS peptide coverage. The sequence segments covered by observed peptides are underlined. Three amino acid residues (Q66, I89, and I245) that do not match the sequence of ORF 12.16 are indicated in blue. (Note that residues 66, 89 and 245 do match one of the other isoforms, see Table 2).

The ORFs derived from the 48,880 transcripts were cross-examined with the program SEQUEST [22] using peptide sequences identified with 2D LC-MS/MS from the purified native enzyme preparation. SEQUEST identified ORF 12.16 as having the highest peptide coverage. As mentioned above, ORF12.16 was one of the 30 sulfotransferase candidate ORFs identified by HMMER. Greater than 90% of the ORF 12.16 amino acid sequence could be matched to peptide sequences derived from the enzyme preparation. ORF 12.16 encodes a 309 amino acid protein with a molecular weight of 35,605 Da. This molecular weight is in agreement with the measured molecular weight of the purified native enzyme. The amino acid sequence of ORF 12.16 and the coverage by MS-determined peptide sequences are shown in Fig 6.

In parallel, PEAKS was used to identify peptide sequences from the 2D LC-MS/MS spectral data. The PEAKS derived peptide sequences differed at only 3 amino acid positions from those encoded by ORF 12.16. PEAKS identified position 66 as an arginine, 89 as lysine and 245 as asparagine.

Among the high coverage ORFs identified by SEQUEST and also by HMMER as sulfotransferases, the two with the next highest LC-MS/MS spectra matches, as compared to 12.16, were ORFs 21753 and 19382, see (Table 1). Additionally, there were two non-sulfotransferase related ORFs which had a high number of hits (about 500 spectra each) and were determined by BLAST analysis to be most similar to a GDP Dissociation Inhibitor with an E value of 0.0 and a molecular weight of 50 kD and to an 2’-5’ oligo-adenylate synthase with an E value of 1e-77 and a molecular weight of 37 kD. The GDP Dissociation Inhibitor is likely the major protein band at about 55 kD visible in the stained protein gel used to isolate the Coel-ST sample for LC-MS/MS (S1 Fig). Despite the effort to exclude the 27 and 55 kD bands from the gel slice, there was likely sufficient contaminating protein in the gel slice used for 2D LC-MS/MS analysis to account for the two non-sulfotransferase ORFs.

**Table 1:** Top 5 ORFs identified by SEQUEST.

Transcript ID #	Number of spectra	MW of ORF	MW of Closest BLAST hit*	BLAST Protein annotation
**12.16**	773	35605	35091	sulfotransferase
21753	93	34329	35091	sulfotransferase
19382	51	36894	36286	sulfotransferase
18969	543	53248	50089	GDP dissociation inhibitor
100402	519	27403	37355	2’-5’ oligo-adenylate synthase

Number of spectra that cover transcriptome derived ORFs, the MW of each ORF-encoded protein, and MW of the highest identity protein from BLAST alignment.

*The top scoring BLAST hit for each of all 5 ORFs against the NCBI non-redundant protein sequence database were from the closely related soft coral, *Dendronephthya gigantea*.

### Coelenterazine sulfotransferase encoding cDNA

**Fig 7:**
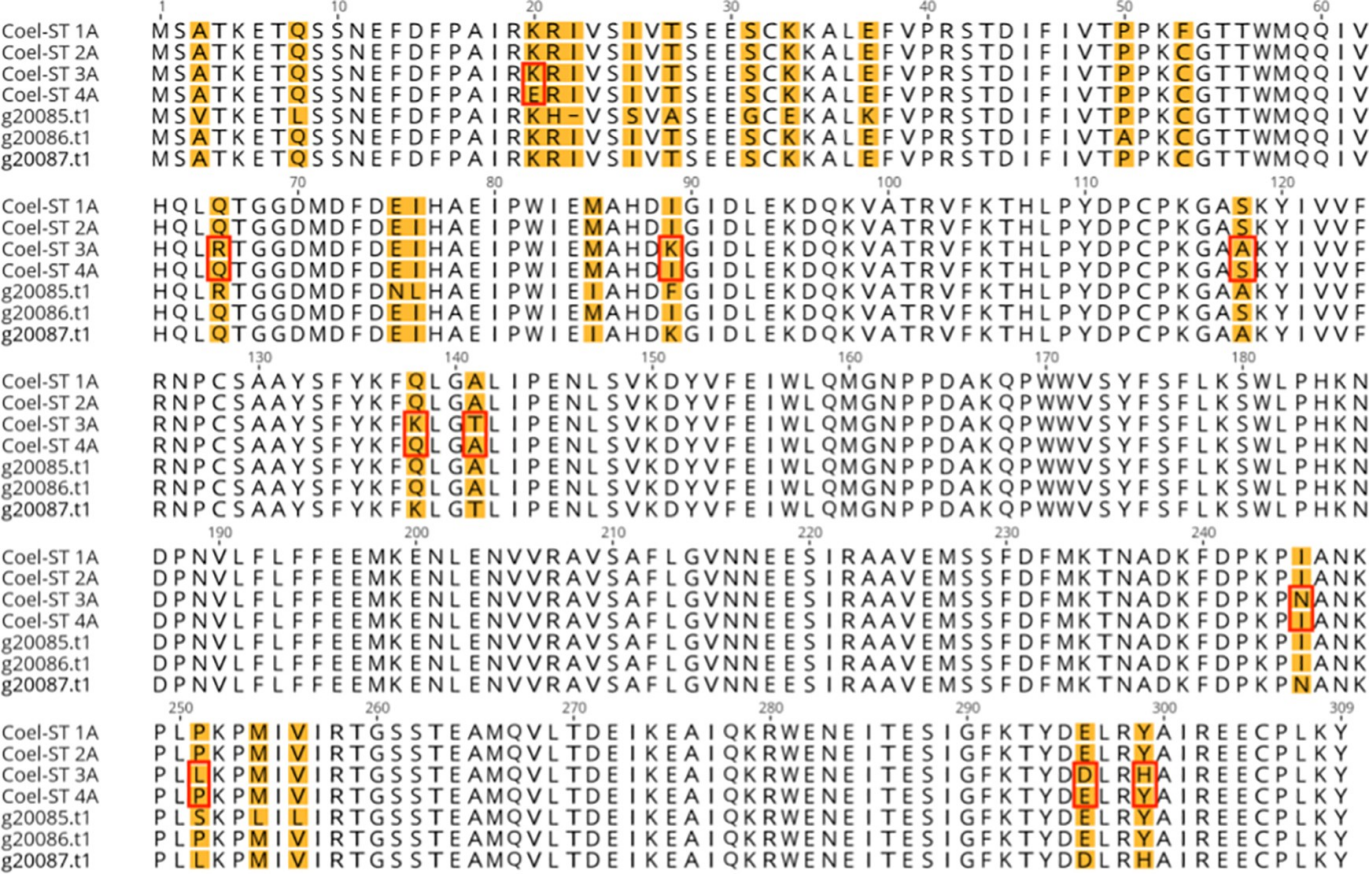
Alignment of the four Coel-ST isoforms, 1A, 2A, 3A and 4A, along with the 3 most closely related predicted proteins g20085.t1, g20086.t1, g20087.t1 from Jiang *et al*. 2019 [23]. The positions that vary among all seven ORFs are highlighted in yellow. The residues that vary between Coel-ST 3A and 4A are outlined in red.

We reasoned that the most likely cDNA to encode the coelenterazine sulfotransferase was the one with the highest peptide coverage, encoded by the 12.16 ORF. It was also identified as a sulfotransferase by HMMER and encoded a protein of the correct molecular weight. The most compelling criterion was the 90% peptide sequence coverage of the ORF. Therefore the 5’ and 3’ terminal nucleic acid sequences of the ORF 12.16 were used as primer sequences for PCR (see Materials and Methods) to amplify the cDNA from the *Renilla* RNA. The amplified cDNA fragment was of the predicted size and was cloned into a plasmid vector (NEB PCR Cloning kit, E1202S) and transformed into *E*. *coli*. The DNA sequence from the four transformants that were analyzed, clearly reflected the sequence of the targeted ORF, further confirming the assembly of the two transcripts constituting ORF12.16. Surprisingly, the sequence of the four transformants represented four different closely related protein sequences. We chose the two most divergent ORFs to express and assess their ability to convert coelenterazine sulfate to coelenterazine. It should be noted that sulfotransferase isoforms 3A and 4A (Fig 7) differ by 10 amino acids that are located throughout the protein. Table 2 lists the amino acid differences of the four ORFs. Three other closely related ORFs can be found in a recent *R*. *muelleri* database of predicted proteins [23] see Fig 7. There were no exact matches between the ORFs found in the published list of predicted proteins and those reported here. All seven ORFs aligned in Fig 7 vary from each other by at least one amino acid residue.

**Table 2:** Amino acid differences in the four different translated isoforms of the 12.16 assembled transcript.

Coel-ST (4A)	1A	2A	3A
E20 C53 Q66 I89 S118 Q128 A141 I245 P251 E296 Y299	K F	K	K R K A K T N L D H

### Recombinant coelenterazine sulfotransferase activity

**Fig 8:**
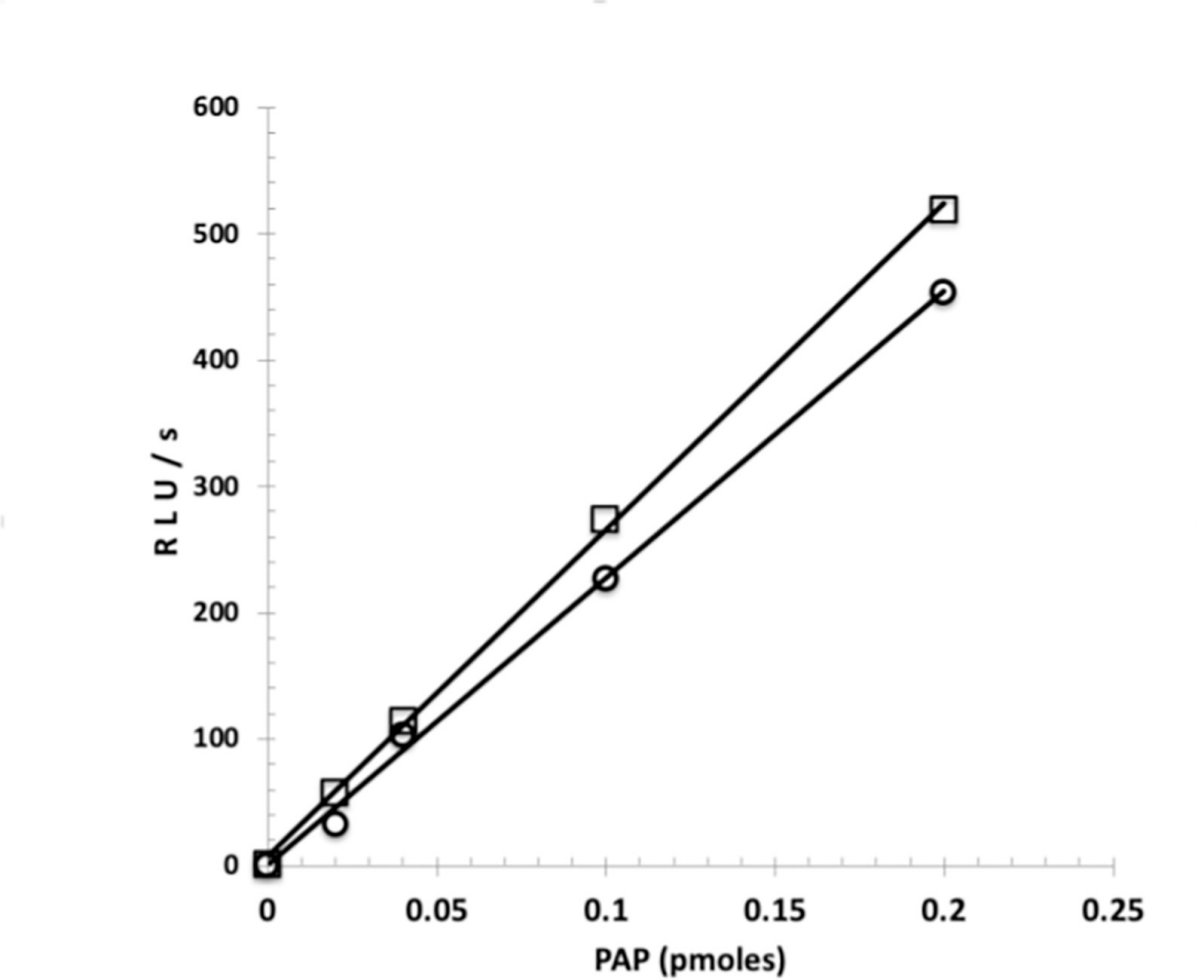
PAP assay. The Coelenterazine sulfotransferase Assay was performed as described in Materials and Methods. The reaction volumes were 100 μl and the amount of PAP varied as shown. The amount of sulfotransferase was 9.5 nmoles for 3A(circles) and 4 nmoles for 4A(squares).

As was shown with the native enzyme preparation the two recombinant isoforms, Coel-ST 3A and 4A, also require PAP (Fig 8) for the conversion of coelenterazine sulfate to coelenterazine. Furthermore as first described by Stanley in 1975 [7] and again by Anderson in 1978 [8] the detection of very low levels of PAP can be achieved in the linked assay with the luciferase. Here we could detect as little as 0.02 picomoles of PAP with either form of sulfotransferase, 3A or 4A and estimated that at these low concentrations of PAP, the specific activity of 4A is 2 to 3 times greater than that of 3A (Fig 8).

The two isoforms differ at 10 amino acid residues distributed throughout the length of the protein (Fig 7 and Table 2). Both forms 3A and 4A convert coelenterazine sulfate to coelenterazine, with form 4A showing approximately 2–3 fold higher specific activity.

### Sulfuryl group transfer from coelenterazine sulfate to PAP by other sulfotransferases

**Fig 9:**
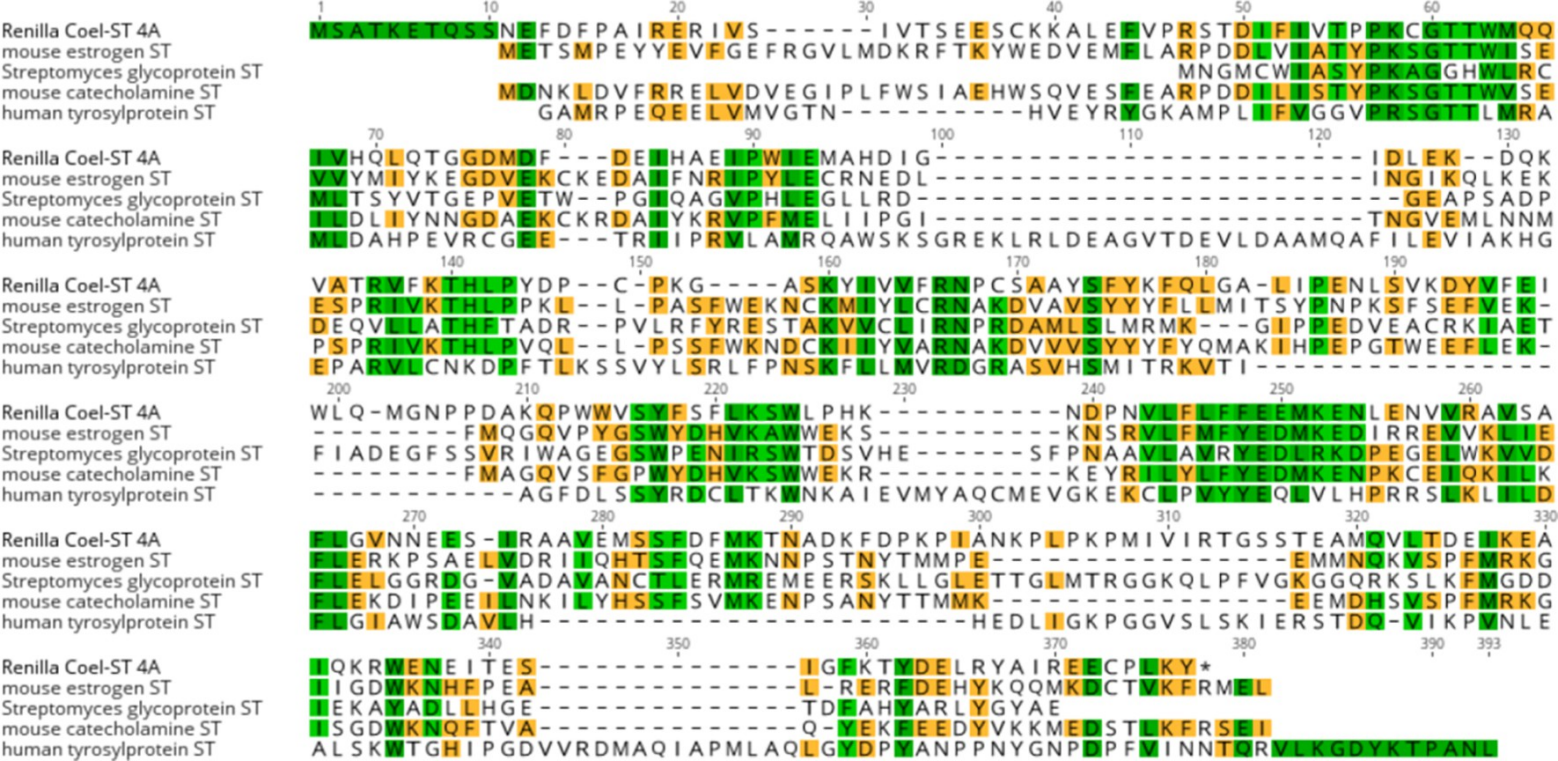
Alignment of five sulfotransferases. Amino acid sequence alignment of *Renilla* Coel_ST 4A with the four sulfotransferases assayed. The sequence similarity of all 5 sulfotransferases is indicated by color highlighting.

Taking advantage of the exquisite sensitivity of the bioluminescent assay, we investigated whether using other sulfotransferases we could detect at some level the removal of the sulfuryl group from coelenterazine sulfate. We therefore selected four sulfotransferases to test for this activity (Fig 9). We expressed and purified from *E*. *coli* the mouse estrogen ST [24], *Streptomyces* glycopeptide ST [25] mouse catecholamine ST [26], and human tyrosylprotein ST [27]. The activity using coelenterazine sulfate as a substrate was measured by the same linked assay used for Coel-ST, i.e., the conversion of the product, coelenterazine, by RLuc to light. Indeed 3 of the 4 other STs had detectable activity with coelenterazine sulfate. We determined that the mouse estrogen ST had the highest relative activity among the non-cognate STs with about 1% of the activity of Coel-ST with coelenterazine sulfate as substrate (S3 Fig).

**Fig 10:**
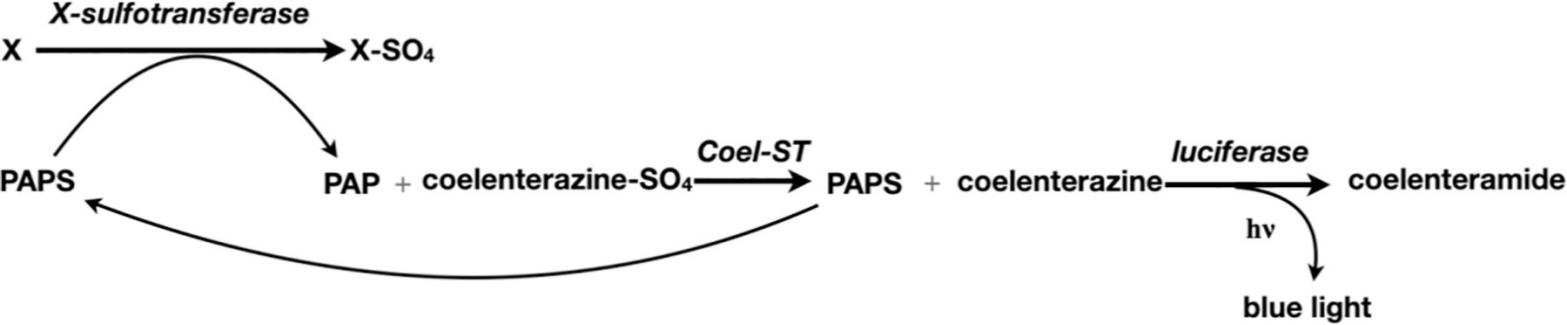
Generalized linked sulfotransferase assay.

In light of the results with the above four STs, we propose and outline here a general assay methodology utilizing Coel-ST that could be used in linked sensitive bioluminescent assays to measure other unrelated sulfotransferase activities (Fig 10). The high sensitivity for the detection of low levels of PAP by Coel-ST is a consequence of the favored direction of the Coel-ST reaction which is the transfer of the sulfuryl group from coelenterazine sulfate to PAP. This is the reverse direction of the majority of other sulfotransferase reactions, where transfer of a sulfuryl group from PAPS to the amino or hydroxyl group of a pharmaceutical compound, carbohydrate, xenobiotic or other acceptor molecule is the physiologically relevant reaction. This distinct property of Coel-ST as well as the high sensitivity of bioluminescent detection offer a unique opportunity to develop assays for other sulfotransferases by coupling them to the Coel-ST PAP assay. When a sulfotransferase has little or no activity towards coelenterazine sulfate, the Coel-ST can be used to measure the activity of an X-sulfotransferase by linking the generation of PAP from the X-ST reaction to the generation of coelenterazine by Coel-ST measured by luminescence generated by *Renilla* luciferase (Fig 10). We expect that this coupling can be applied to a large variety of sulfotransferase assays which would eliminate the costly and hazardous use of ^35^S [28] and enable high throughput assays for enzyme inhibitors.

## Discussion

In this report, *Renilla* coelenterazine sulfotransferases were identified, cloned, sequenced and expressed. This was achieved by using two complementary technologies: the generation of a *de novo* assembled transcriptome and peptide sequencing of an isolated target protein. When a genome sequence and its annotation are not available, it is shown here that intersecting a protein sequence and a transcriptome is an effective method for obtaining the coding sequence of a specific protein. The coelenterazine sulfotransferase protein was purified from *R*. *muelleri* and peptide sequences were determined by 2D LC MS/MS. In parallel, RNA was isolated from *Renilla* from which a cDNA library was prepared and sequenced on the Illumina platform and the data used to generate an assembled transcriptome was translated *in silico*. Candidate RNA transcripts that closely matched the peptide sequences obtained from the purified sulfotransferase were cloned with transcript-specific primers as cDNAs. Sequencing of the isolated cDNA clones indicated that two of the isoforms of the Coel-ST differ by 10 amino acids. Both isoforms (3A and 4A) were expressed and shown to encode active sulfotransferase that catalyzes the conversion of coelenterazine sulfate to coelenterazine. A recent genomic study of *R*. *muelleri* [23] has generated a proteome of predicted proteins in which we identified 3 close homologs to the sulfotransferases identified here, the closest varied by only one amino acid residue (Fig 7).

In another bioluminescence related sulfotransferase system report from 2019, a sulfotransferase for the chemically distinct firefly luciferin has been identified and cloned from fireflies [29]. The firefly luciferin sulfotransferase was shown to catalyze the formation of the storage form, luciferin sulfate, and proposed to catalyze the activation of luciferin sulfate to luciferin at high PAP concentration. In contrast, the different isoforms of the Coel-ST we identified from *Renilla* may account for either creating the storage form, coelenterazine sulfate, or conversion of the storage form to coelenterazine.

It is generally understood that sulfotransferases transfer a sulfuryl group from the donor PAPS to acceptor alcohol or amine to form the corresponding sulfated acceptor. It is recognized that sulfotransferase reactions are reversible and under appropriate *in vitro* conditions sulfotransferases have been used to generate PAPS from PAP [30]. However, *in vivo* the removal of the sulfuryl group from acceptor sulfate usually proceeds via a sulfatase [31] resulting in alcohol acceptor and sulfate ion. For example, in humans the removal of the sulfuryl group from estradiol sulfate utilizes a sulfatase [32]. The levels of estradiol are exceedingly low, pM, and conserving the sulfate in the form of PAPS may not be of any significant value. On the other hand, for *Renilla*, because of its relatively large cache of coelenterazine sulfate, it may be more advantageous to retain the sulfate in the form of PAPS, in order to reduce the need to assimilate sulfate for sulfation of the newly synthesized (or scavenged) coelenterazine. Cormier (1962) [14] reported that bioluminescence in *Renilla* is dependent on PAP, indicating that the removal of the sulfuryl group from coelenterazine sulfate takes place via a sulfotransferase, generating PAPS. Consistent with this observation, no *Renilla* sulfatases that act on coelenterazine have been reported. The conservation of assimilated sulfate is of value because of the high cost of ATP to generate PAPS *de novo* from sulfate with ATP sulfurylase and APS kinase, thus the recycling of PAPS/PAP with the sulfotransferase reaction confers conservation of energy; it alleviates the need of additional energy input (ATP) to generate PAPS which would be required if a sulfatase were involved in activating the coelenterazine sulfate as shown below.


$$ATP\;sulfurylase\;reaction:\;SO_{4}{}^{2-}+ATP\to APS+PP_{i}$$

APSkinasereaction:APS+ATP→PAPS+ADP

**Fig 11:**
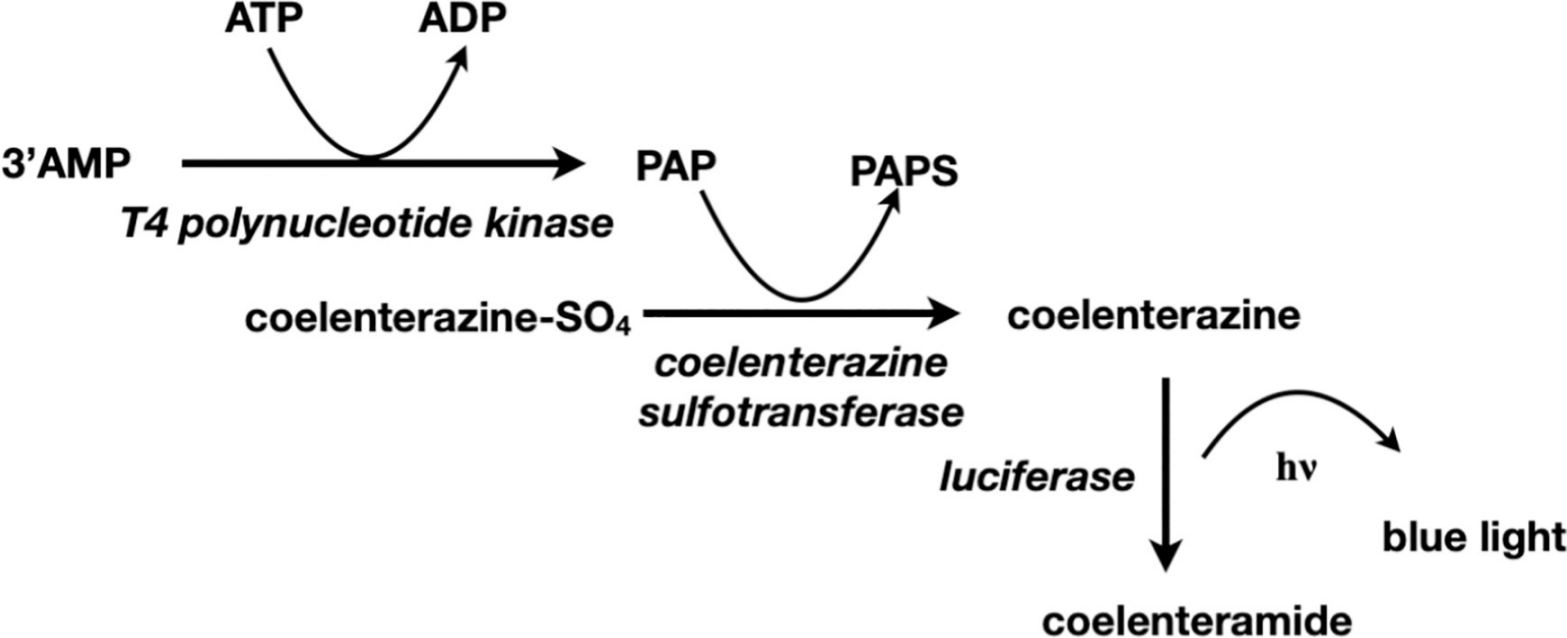
ATP bioluminescence assay using Coel-ST in a three-enzyme cascade.

By obtaining the sequence of the *Renilla* Coel-ST, the highly sensitive PAP measurement assay described previously [7] is now considerably more accessible by using a recombinant sulfotransferase (F 8). We considered that we may be able to also detect low levels of ATP with a linked three enzyme cascade. The basis of this assay is the coupling of T4 polynucleotide kinase (3’ phosphatase minus), coelenterazine sulfotransferase and *Renilla* luciferase reactions (Fig 11).

The cascade initiates with T4 polynucleotide kinase transferring a phosphate from ATP to 3’ AMP to form PAP (3’-phosphoadenosine 5’-phosphate) [33]. The PAP is then used to accept the sulfuryl group from the coelenterazine sulfate, thereby activating it for conversion to a bioluminescent signal with the luciferase. The amount of bioluminescence produced corresponds to the ATP concentration (see S4 Fig).

## Supporting information

S1 Fig PAGE of sulfotransferase preparation. Lane b is the final enzyme preparation. Lane a is a protein molecular weight standard. The gel was stained with Coomassie Blue.
(DOCX)

S2 Fig Coel-ST PAP dependence. Luminometer output (RLUs per second) versus time at the indicated amounts of PAP. Reaction conditions as described in Materials and Methods, PAP assay.
(DOCX)

S3 Fig Comparing STs. 100 μl reactions containing 100 mM Bis-tris propane pH 7.0, 1 mM DTT, 1 mM EDTA, 20 μM PAP, 200 nM coelenterazine sulfate, and 10 nM RLUC. 100 μls were aliquoted into 96 well plates and incubated at 25°C for one minute to ensure the background signal was stable. Then the reaction was initiated by the addition of 2 μg of purified sulfotransferase and activity monitored in a Centro LB 960 luminometer (Berthold) plate reader at 25°C, by measuring relative light units per 2 second (RLU/s). The sulfotransferase proteins were obtained by expressing them in *E*. *coli* and purified by affinity chromatography via their amino terminal His-tags.
(DOCX)

S4 Fig ATP assay. The top panel shows luminometer output (RLUs over time) at the indicated amounts of ATP. The bottom panel has an expanded scale to better visualize the reactions with the lowest amounts of ATP. Zero ATP represents the level of background signal. Each reaction was 100 μl containing 70 mM Tris-HCl pH 7.6, 10 mM MgCl_2_, 5 mM DTT, 100 mM NaF, 100 μM 3’-AMP, 125 nM coelenterazine sulfate, 2 μl native Coel-ST (corresponding to approximately 1% of partially purified Coel-ST from 1 *Renilla* animal), 10 nM RLuc, 10 units T4 Polynucleotide Kinase (3’ phosphatase minus, NEB M0236), and the indicated amount of ATP. The reactions were initiated by the addition of T4 polynucleotide kinase at 25°C. Relative light units per second (RLU/s) were measured in a Centro LB 960 luminometer (Berthold) plate reader over a 35-minute time course.
(DOCX)

S1 Table Sulfuryl group transfer from coelenterazine sulfate to PAP by other sulfotransferases. We surveyed four sulfotransferases (STs) for their ability to transfer the sulfate from coelenterazine sulfate to PAP producing coelenterazine. The mouse estrogen ST was chosen based on sequence similarity (Fig 9) to the Coel-ST and the others based on the range of molecular weights of their cognate substrates.
(DOCX)
