# Supplementary material for: Coelenterazine sulfotransferase from Renilla muelleri
Source: PLoS One. 2022 Oct 17;17(10):e0276315. doi: 10.1371/journal.pone.0276315 (PMC9576082; doi:10.1371/journal.pone.0276315)
Supplement: S1 Fig — Lane b is the final enzyme preparation. Lane a is a protein molecular weight standard. The gel was stained with Coomassie Blue. (DOCX) [file pone.0276315.s001.docx]

**Protein Gel**


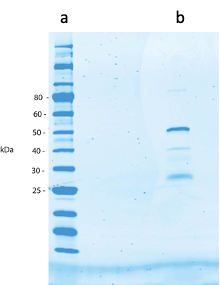


**S1 Fig. PAGE of sulfotransferase preparation.** Lane b is the final enzyme preparation. Lane a is a protein molecular weight standard. The gel was stained with Coomassie Blue.
