# Supplementary material for: Coelenterazine sulfotransferase from Renilla muelleri
Source: PLoS One. 2022 Oct 17;17(10):e0276315. doi: 10.1371/journal.pone.0276315 (PMC9576082; doi:10.1371/journal.pone.0276315)
Supplement: S2 Fig — Luminometer output (RLUs per second) versus time at the indicated amounts of PAP. Reaction conditions as described in Materials and Methods, PAP assay. (DOCX) [file pone.0276315.s002.docx]

## Coel-ST PAP dependence


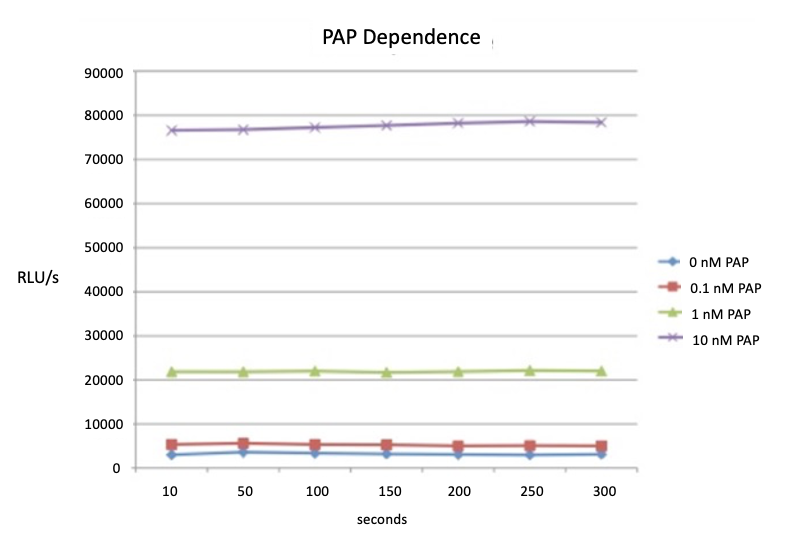


**S2 Fig. Coel-ST PAP dependence.**

Luminometer output (RLUs per second) versus time at the indicated amounts of PAP.

Reaction conditions as described in Materials and Methods, PAP assay.
