# Supplementary material for: Coelenterazine sulfotransferase from Renilla muelleri
Source: PLoS One. 2022 Oct 17;17(10):e0276315. doi: 10.1371/journal.pone.0276315 (PMC9576082; doi:10.1371/journal.pone.0276315)
Supplement: S3 Fig — 100 μl reactions containing 100 mM Bis-tris propane pH 7.0, 1 mM DTT, 1 mM EDTA, 20 μM PAP, 200 nM coelenterazine sulfate, and 10 nM RLUC. 100 μls were aliquoted into 96 well plates and incubated at 25°C for one minute to ensure the background signal was stable. Then the reaction was initiated by the addition of 2 μg of purified sulfotransferase and activity monitored in a Centro LB 960 luminometer (Berthold) plate reader at 25°C, by measuring relative light units per 2 second (RLU/s). The sulfotransferase proteins were obtained by expressing them in E. coli and purified by affinity chromatography via their amino terminal His-tags. (DOCX) [file pone.0276315.s003.docx]

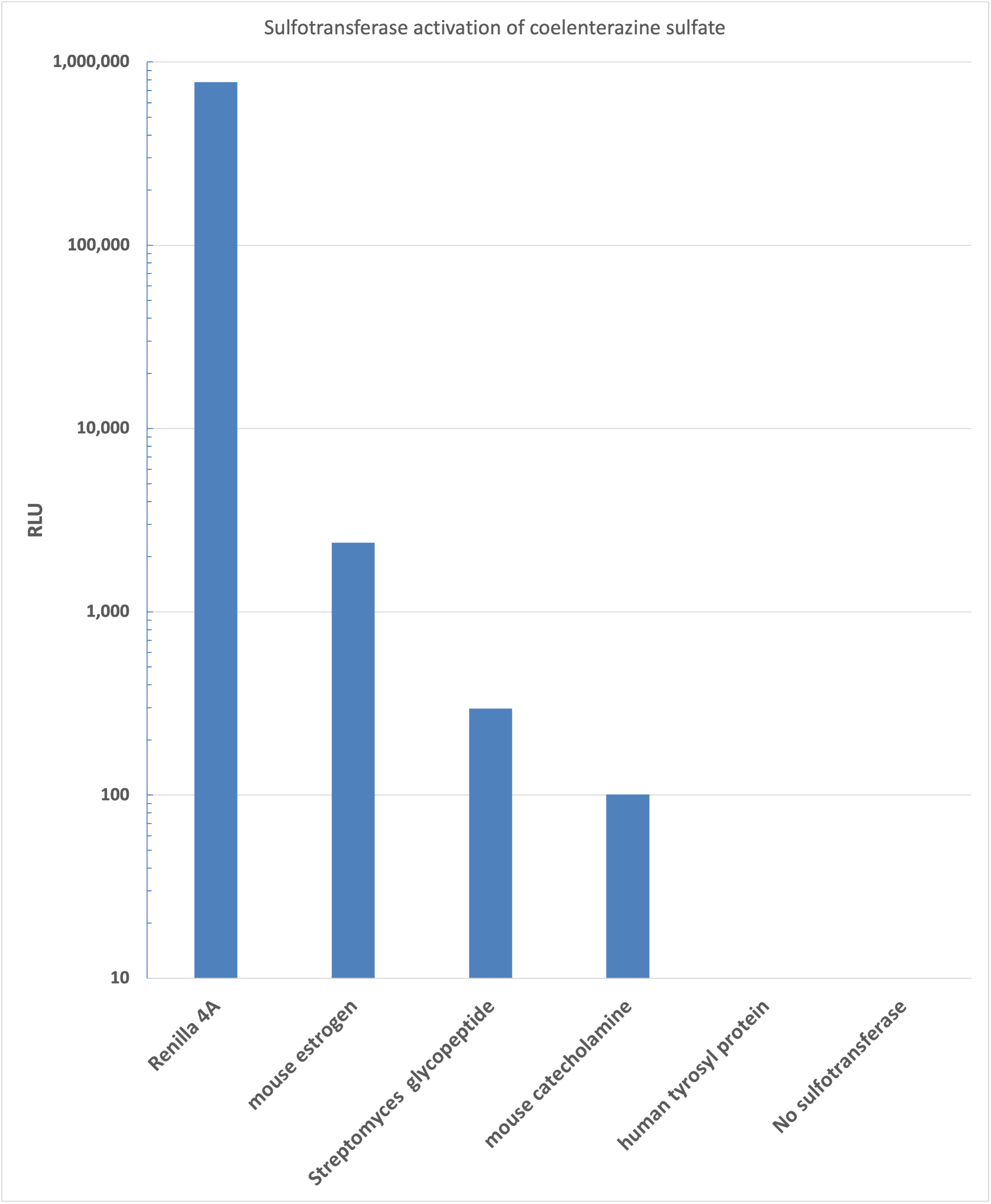


**S3 Fig. Comparing STs.**

100 µl reactions containing 100 mM Bis-tris propane pH 7.0, 1 mM DTT, 1mM EDTA, 20 µM PAP, 200 nM coelenterazine sulfate, and 10 nM RLUC. 100 uls were aliquoted into 96 well plates and incubated at 25^o^ C for one minute to ensure the background signal was stable. Then the reaction was initiated by the addition of 2 µg of purified sulfotransferase and activity monitored in a Centro LB 960 luminometer (Berthold) plate reader at 25^o^ C, by measuring relative light units per 2 second (RLU/s). The sulfotransferase proteins were obtained by expressing them in *E. coli* and purified by affinity chromatography via their amino terminal His-tags.
