# Supplementary material for: Coelenterazine sulfotransferase from Renilla muelleri
Source: PLoS One. 2022 Oct 17;17(10):e0276315. doi: 10.1371/journal.pone.0276315 (PMC9576082; doi:10.1371/journal.pone.0276315)
Supplement: S4 Fig — The top panel shows luminometer output (RLUs over time) at the indicated amounts of ATP. The bottom panel has an expanded scale to better visualize the reactions with the lowest amounts of ATP. Zero ATP represents the level of background signal. Each reaction was 100 μl containing 70 mM Tris-HCl pH 7.6, 10 mM MgCl2, 5 mM DTT, 100 mM NaF, 100 μM 3’-AMP, 125 nM coelenterazine sulfate, 2 μl native Coel-ST (corresponding to approximately 1% of partially purified Coel-ST from 1 Renilla animal), 10 nM RLuc, 10 units T4 Polynucleotide Kinase (3’ phosphatase minus, NEB M0236), and the indicated amount of ATP. The reactions were initiated by the addition of T4 polynucleotide kinase at 25°C. Relative light units per second (RLU/s) were measured in a Centro LB 960 luminometer (Berthold) plate reader over a 35-minute time course. (DOCX) [file pone.0276315.s004.docx]

## ATP ASSAY


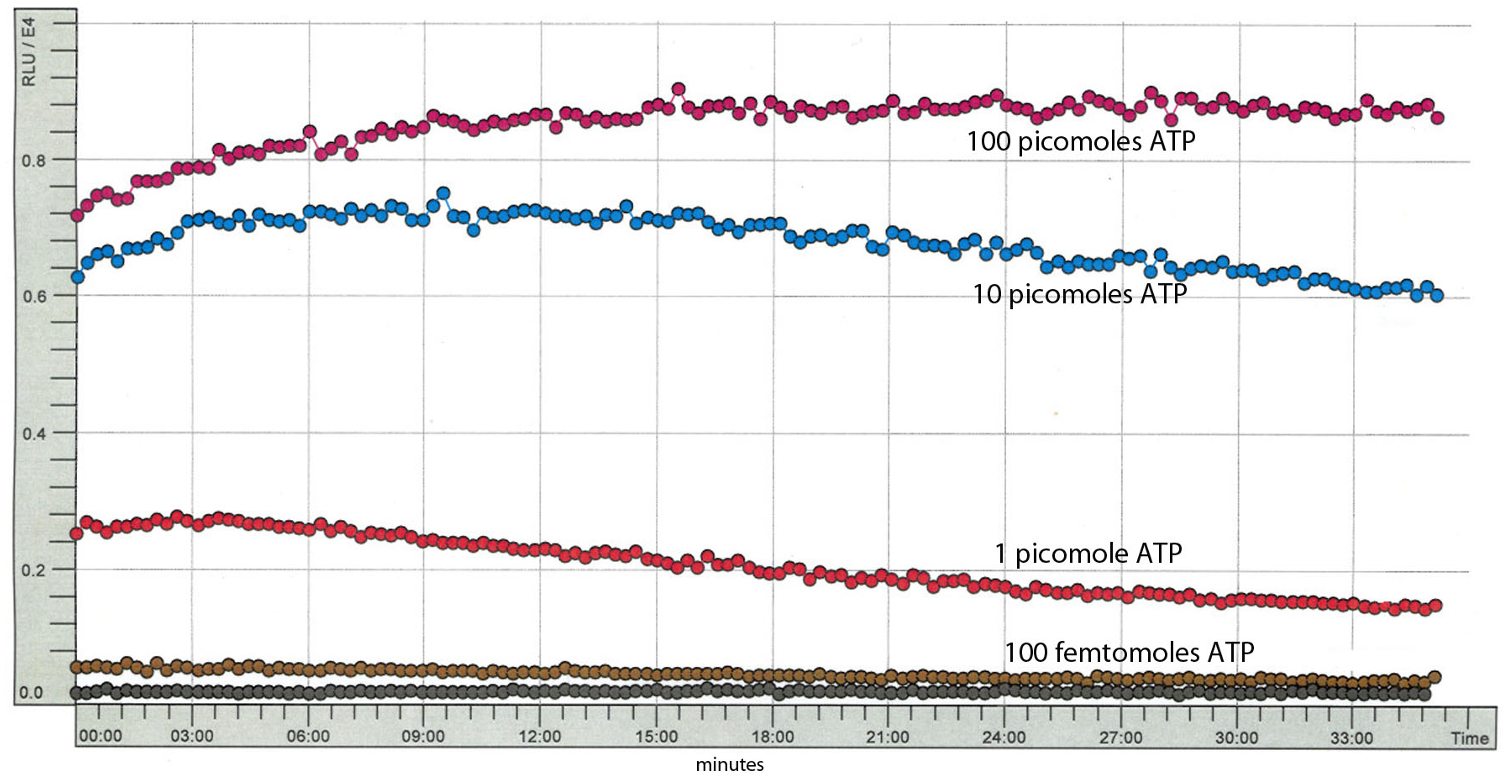


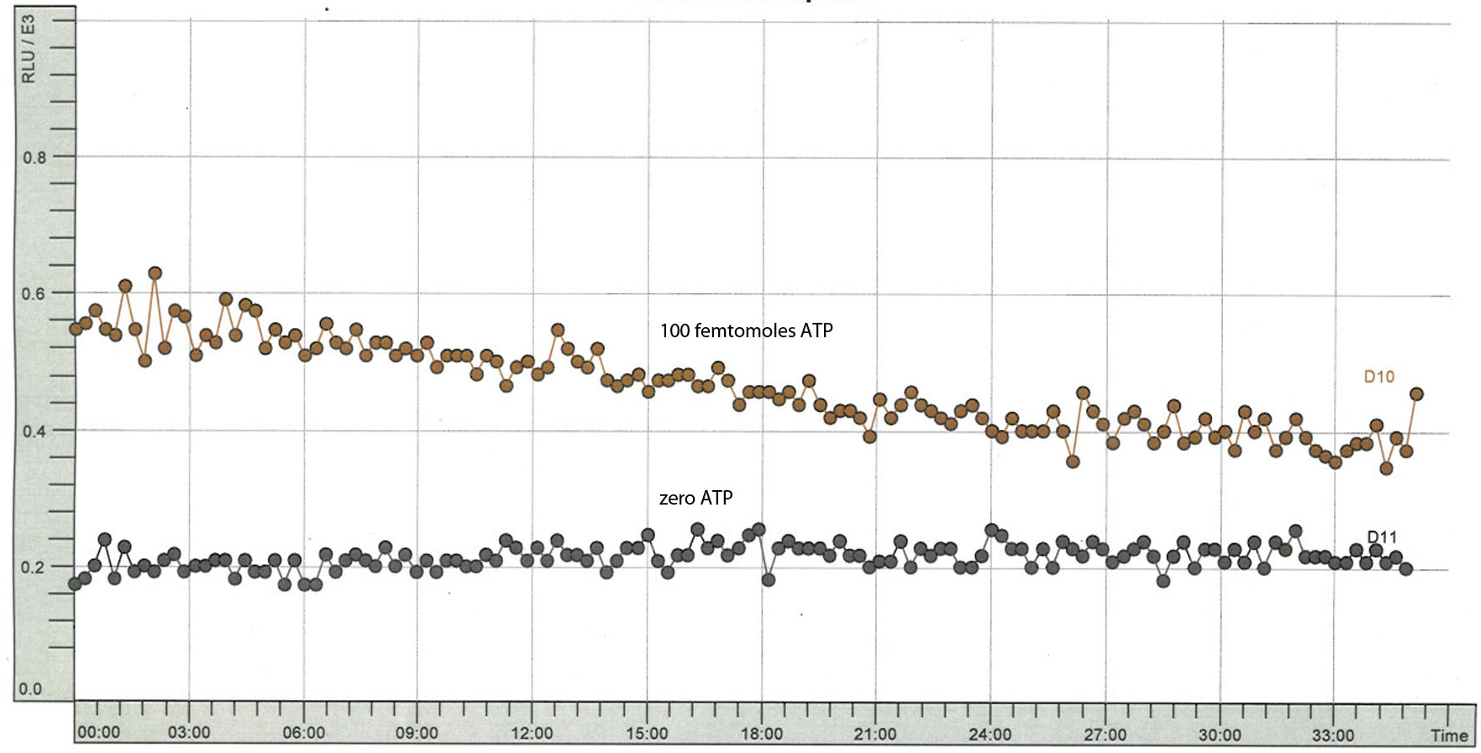


**Fig S4. ATP Assay.**

The top panel shows luminometer output (RLUs over time) at the indicated amounts of ATP. The bottom panel has an expanded scale to better visualize the reactions with the lowest amounts of ATP. Zero ATP represents the level of background signal.

Each reaction was 100 µl containing 70 mM Tris-HCl pH 7.6, 10 mM MgCl_2_, 5 mM DTT, 100 mM NaF, 100 µM 3' AMP, 125 nM coelenterazine sulfate, 2 µl native Coel-ST (corresponding to approximately 1% of partially purified Coel-ST from 1 *Renilla* animal), 10 nM RLuc, 10 units T4 Polynucleotide Kinase (3' phosphatase minus, NEB M0236), and the indicated amount of ATP. The reactions were initiated by the addition of T4 polynucleotide kinase at 25^o^ C. Relative light units per second (RLU/s) were measured in a Centro LB 960 luminometer (Berthold) plate reader over a 35 minute time course.
