# Supplementary material for: Coelenterazine sulfotransferase from Renilla muelleri
Source: PLoS One. 2022 Oct 17;17(10):e0276315. doi: 10.1371/journal.pone.0276315 (PMC9576082; doi:10.1371/journal.pone.0276315)
Supplement: S1 Table — We surveyed four sulfotransferases (STs) for their ability to transfer the sulfate from coelenterazine sulfate to PAP producing coelenterazine. The mouse estrogen ST was chosen based on sequence similarity (Fig 9) to the Coel-ST and the others based on the range of molecular weights of their cognate substrates. (DOCX) [file pone.0276315.s005.docx]

## S1 Table

## Sulfuryl group transfer from coelenterazine sulfate to PAP by other sulfotransferases.

We surveyed four sulfotransferases (STs) for their ability to transfer the sulfate from coelenterazine sulfate to PAP producing coelenterazine (Table S1). The mouse estrogen ST was chosen based on sequence similarity (Fig 9) to the Coel-ST and the others based on the range of molecular weights of their cognate substrates.

| **Sulfotransferase** | **PDB code** | **Relative rate *** | **Cognate substrate**  **(MW in Daltons)** |
| --- | --- | --- | --- |
| **Renilla Coel-ST 4A** | - | 1 | coelenterazine (424) |
| **mouse estrogen ST** | 1AQU | 0.002 | estradiol (272) |
| **Streptomyces glycopeptide ST** | 2OVB | 0.0006 | desulfoA47934 (1233) |
| **mouse catecholamine ST** | 2ZPT | 0.00006 | dopamine (153) |
| **human tyrosylprotein ST** | 3AP1 | <0.00001 | peptide (~ 4000) |
| **NO ST** | - | <0.00001 |  |

* with coelenterazine sulfate as substrate
